# Contingent Negative Variation and Working Memory Maintenance in Adolescents with Low and High Motor Competencies

**DOI:** 10.1155/2018/9628787

**Published:** 2018-04-18

**Authors:** Sebastian Ludyga, Christian Herrmann, Manuel Mücke, Christian Andrä, Serge Brand, Uwe Pühse, Markus Gerber

**Affiliations:** ^1^Department of Sport, Exercise and Health, University of Basel, Basel, Switzerland; ^2^Department of School Sport, Leipzig University, Leipzig, Germany; ^3^Center for Affective, Stress and Sleep Disorders, Psychiatric Clinics of the University of Basel, University of Basel, Basel, Switzerland; ^4^Substance Abuse Prevention Research Center and Sleep Disorders Research Center, Kermanshah University of Medical Sciences (KUMS), Kermanshah, Iran

## Abstract

Although it has been suggested that motor and cognitive development is interrelated, the link between motor competencies and neurophysiological indices of working memory operations has not yet been examined in adolescents. This study is aimed at comparing contingent negative variation and working memory performance between adolescents with low and high motor competencies. In eighty-two adolescents, motor competencies were assessed with the MOBAK-5 (basic motor competencies, 5th grade) test battery and a median split was performed on this variable to divide them into low and high performers. Additionally, all participants completed a Sternberg paradigm to assess working memory maintenance. The initial (iCNV) and terminal (tCNV) components of the contingent negative variation elicited by the cognitive task were recorded using electroencephalography. Higher working memory maintenance was found in adolescents with high motor competencies compared to those with low motor competencies. Cluster-based permutation testing further revealed increased iCNV in adolescents with higher motor competencies. In contrast, there was no difference in tCNV between groups. The findings suggest that high working memory maintenance and effective task preparation are both linked to high motor competencies. Thus, high performers on complex motor tasks seem to rely more on a proactive control strategy, which is optimal in tasks with high working memory demands.

## 1. Introduction

Working memory refers to short-term maintenance, storage, and manipulation of sensory information [1]. Evidence from neuroimaging studies suggests that working memory tasks recruit the frontoparietal network, which is involved in top-down modulation of cognitive processing [2]. This indicates that working memory underpins a wide range of behaviors, spanning from perception to higher-order cognition and action control [3]. Further, working memory is considered crucial for academic achievement [4], which is partly due to its positive association with reading comprehension [5] and math skills at school age [6]. Thus, the promotion of working memory is regarded as an important target for interventions in children and adolescents.

A recent review suggests that enhancements of working memory following cognitive training are associated with changes in the frontoparietal network and basal ganglia [7]. Similarly, aerobic fitness has been related to both increased volume of these specific regions [8] and higher working memory capacity [9]. To enhance performance in this cognitive domain, physical activity programs have aimed for improvements in quantitative aspects of motor skills (product-oriented assessments) and aerobic fitness in particular. However, recent meta-analytical findings suggest that enriched physical activity programs, which are characterized by higher coordinative and cognitive demands, elicited even greater benefits for working memory than pure aerobic training [10]. As those interventions are expected to benefit motor competencies, this is a first indication that such qualitative aspects of motor skills (process-oriented assessments) may also be related to working memory.

Motor competencies are understood as fundamental skills (including object control and locomotor skills) that enable goal-directed human movement [11]. These competencies are developed during childhood and refined into context- and sport-specific skills in subsequent years [12]. As working memory and its underlying neural circuitry also continue to develop gradually until adolescence [13], the acquisition of motor proficiency and the maturation of such higher-order cognitive skills appear to be interrelated [14]. Findings from neuropsychology further indicate that motor skills and cognitive control share common neural substrates. Based on a recent review, the prefrontal cortex, the cerebellum, and the basal ganglia interact to guide behavior in complex cognitive and movement tasks [15], which demand both anticipation and prediction. Similarly, an overview of cross-sectional studies supports this association on a behavioral level, although far more evidence was provided for children compared to adolescents [16].

The evidence that supports a link between motor competencies and working memory in particular is mainly based on the examination of end-state processes occurring after a series of physiological events [17–19]. Consequently, it remains unclear how motor competencies relate to the subtle aspects of cognitive processing that contribute to working memory. Preparation, expectation, and anticipation allow the optimization of resources for an upcoming event and are crucial in both working memory tasks and complex movements [20]. Using event-related potentials (ERPs), neurophysiological indices of such task preparation processes can be examined within a period between the encoding of a warning stimulus (S1) and a stimulus that requires a response (S2). The Sternberg task [21] uses such a paradigm and therefore allows the investigation of preparation processes. This task requires participants to remember a set of items (S1), for example, an array of letters. Following a brief delay, a probe (S2) is presented and participants have to indicate whether it was part of the set.

The S1-S2 interval of the Sternberg task has been found to elicit a contingent negative variation [22, 23], which is a centrally distributed negative-going slow potential [20]. This component is associated with the integration of sensory information and the organization of a response [24]. If the time period between S1 and S2 is of sufficient length (≥1500 ms), the initial (iCNV) and terminal (tCNV) components of the CNV can be differentiated [22, 25, 26]. While the iCNV elicited by S1 reflects an orienting response and early-response selection [27], the tCNV is related to stimulus anticipation and/or response preparation at S2 [28]. Both components share similar neural generators, with the activity in frontoparietal networks as well as in premotor-motor and sensory areas being the main contributors to the elicitation of the CNV [24, 29]. As the amplitude of the CNV increases throughout childhood and adolescence, this component seems to be sensitive to developmental changes [24]. Previous studies have suggested that lower iCNV amplitude in children relative to adults is partly due to inefficient cue orienting and an immaturity of the distributed prefrontal cortex system [26, 30]. Evidence, albeit limited, suggests that the iCNV is sensitive to physical activity. In children, higher iCNV amplitudes during a Sternberg task were found along with improved accuracy following a 9-month exercise program that aimed for improvements in aerobic fitness [22]. Focusing on both qualitative and quantitative aspects of motor skills, Ludyga et al. showed that, in adolescents, combined aerobic and coordinative training elicited decreases in reaction time and increases in the iCNV following an intervention period of 8 weeks [23]. In both studies, there was no change of the tCNV recorded from the Sternberg task, suggesting that the observed improvements in working memory were partly due to a more efficient orienting response. As behavioral performance on higher-order cognitive tasks has been linked with qualitative aspects of motor skills [16], it seems likely that adolescents with high motor competencies may show greater iCNV amplitude during working memory tasks than peers with low motor competencies. So far, neurophysiological indices and those related to preparation processes in particular have not been compared between high and low performers on tasks demanding motor competencies. However, new insights are necessary to gain an understanding of the mechanisms underlying the relationship of working memory and qualitative aspects of motor skills.

The purpose of this study was to compare the CNV and working memory performance between adolescents with low and high motor competencies. Based on van der Fels et al.'s review [16], we assumed that high performers on complex motor tasks would show greater working memory performance than low performers. Additionally, we expected higher iCNV, but not tCNV, in adolescents with high motor competencies [22, 23].

## 2. Materials and Methods

### 2.1. Participants

For the current investigation, pretest data from two experimental studies were combined [23, 31]. The total sample comprised 82 adolescents (34 females, 48 males) from three private schools with a similar educational level. Eligible students had to have right hand dominance according to the Edinburgh Handedness Inventory [32] and corrected or normal vision and to be 10 to 15 years of age. Participants undergoing pharmacological treatment and those receiving special education services were excluded. Additionally, the prevalence of any acute or chronic diseases, which could possibly restrict physical activity during everyday life, also led to exclusion. Within one week, all participants completed the MOBAK-5 (basic motor competencies, 5th grade) test battery and a modified Sternberg task. By using electroencephalography, ERPs were recorded during the task. Additionally, physical activity levels were objectively measured with accelerometers over seven consecutive days. Participants also filled in a self-report version of the Strengths and Difficulties Questionnaire [33], which is a tool for the assessment of different dimensions of psychopathology (emotional problems, conduct problems, problems with peers, and hyperactivity). These variables were collected as possible covariates, because previous studies have shown a relationship with executive function [9, 34]. Written assent and informed consent were provided by adolescents and their legal guardians, respectively. For both experimental studies, from which data was combined, procedures were approved by the local ethics committee. Moreover, the study protocol followed the ethical principles outlined in the Declaration of Helsinki and its later amendments.

### 2.2. Cognitive Task

Cognitive testing was performed in a separate room with one participant at a time. Following the explanation of the testing procedure, participants were seated in a comfortable chair and the surrounding noise was kept to a minimum. For the assessment of working memory maintenance, a modified Sternberg task was administered with E-Prime 2.0 (PST, USA). During the task, a memory set containing an array of 3 white and 2 green uppercase consonants (S1) was presented focally for 2500 ms on a black background. Participants were instructed to remember the white letters and to ignore the green letters. Following a delay of 2000 ms, a single probe letter (S2) was presented for 250 ms (e.g., ??k??). When the probe appeared, participants had to indicate the presence or absence of the letter in the set by pressing a button on a serial response box with the right or left thumb, respectively. Green letters were never used as probes and only served as distractors to induce additional executive function demands [35]. Prior to each trial, a fixation cross was presented for 1700 to 2200 ms (random variation). Participants completed a practice round with 10 trials, followed by two blocks with 50 trials each (with a 30 s break in between). The presentation of present and absent probes was equiprobable, and the order of the trials was randomized. Mean reaction times obtained from response-correct trials and accuracy were calculated as dependent variables. Depending on the individual performance level, the Sternberg task took 15 to 20 min.

### 2.3. EEG

The electroencephalogram (EEG) was acquired with 64 electrodes using a HydroCel Geodesic Sensor Net (EGI, USA) and amplified by a Net Amps 300 (EGI, USA). As recommended for high-impedance systems, scalp electrode impedances were maintained below 50 kΩ [36]. Recordings were digitized at 250 Hz and referenced to the vertex channel. EEG data was processed offline using BESA Research 6.1 (Brain Electric Source Analysis, Germany). First, blinks and eye movements were detected in continuous data using HEOG and VEOG channels. These ocular artefacts were submitted to automatic adaptive artefact correction, which employs principal component analysis for the separation of artefact components. Second, the remaining artefacts in the period from −2300 ms to S1 onset were rejected based on individual amplitude (134 ± 15 *μ*V) and gradient (64 ± 9 *μ*V) thresholds. Subsequently, a high-pass filter (0.3 Hz; 6 dB/octave) and baseline correction (using the interval from −2200 ms to S1 offset) were applied on recorded data. Following the averaging procedure, the resulting segments were low-pass filtered (30 Hz; 24 dB/octave) and rereferenced to average mastoids. For the analysis of the contingent negative variation, ERP waves were exported for the period from S1 offset to S2 onset.

### 2.4. Motor Competencies

Motor competencies were assessed using the validated MOBAK-5 test instrument [37, 38], which comprises eight different motor tasks related to either object control (bouncing, dribbling, throwing, and catching) or locomotor skills (balancing, rolling, jumping, and running). Participants' motor competencies were assessed by trained test leaders and in groups of three to four adolescents. Prior to testing, the individual motor tasks were explained and demonstrated. Except for throwing and catching, participants had two attempts to accurately perform each task. Successful attempts were scored with one point, resulting in a range of 0 (no successful attempt) to 2 points (2 successful attempts) per task. For throwing and catching, participants had six attempts and scoring was as follows: 0–2 hits = 0 points, 3-4 hits = 1 point, and 5-6 hits = 2 points. The total score was calculated as the sum of the results on all tasks, resulting in a range from 0 to 16 points.

### 2.5. Physical Activity

Participants' physical activity was monitored with triaxial accelerometers (wGT3X-BT, Actigraph, USA) worn on the wrist over seven consecutive days. Datasets were included if participants had at least three valid week days and one valid weekend day, whereby valid was defined by 70% or more wear time per day [39]. Light, moderate, and vigorous physical activity was differentiated based on the algorithm provided by Mattocks et al. [40].

### 2.6. Statistics

Statistical analyses of anthropometric and behavioral data were performed with SPSS 25.0 (IBM, USA). In advance, participants were divided into adolescents with low and high motor competencies using median split, because no normative data exists for MOBAK-5 scores. Based on the recommendation of Iacobucci et al. [41], preliminary assessments of multicollinearity between motor competencies and possible confounders were used to verify that this method allows the examination of group differences with complete analytical integrity. To examine whether or not the percentage of participants in the high and low motor competence groups differed by gender or school, the *χ* ^2^ test was applied. Subsequently, one-way ANOVAs were employed to compare the number of segments included for analysis, anthropometric data, psychopathology, and physical activity between groups. In case of unequal distributions and/or significant group differences, the corresponding variables were entered as covariates in the following comparison. The effect of motor competence on working memory maintenance was examined by applying a MAN(C)OVA with group as between-subjects factor on behavioral performance (reaction time and accuracy). Multivariate main effects and/or interactions were further investigated using univariate ANOVAs. For all statistical comparisons, an alpha level of *p* ≤ 0.05 was considered significant.

ERPs were analyzed with BESA Statistics 2.0 (Brain Electrical Source Analysis, Germany), which employs both parametric and cluster-based permutation testing. This approach has been recommended to deal with the multiple comparison problem arising in electrophysiological studies, because it does not make auxiliary assumptions and allows the use of a statistical test that is maximally sensitive to the expected effect [42]. Preliminary correlational analyses were examined if covariates, which were entered for the main comparison on behavioral performance, also had an effect on the ERP waveforms. In case those variables also influenced the ERP waveforms, subsequent comparisons used *F*-tests with covariates. Otherwise, two-tailed *t*-tests were applied to examine differences between groups. Each comparison followed a two-stage approach. First, parametric tests were applied to identify clusters in time and space where the ERPs differed between groups. Similar to previous studies [22, 23], the latency ranges from −1500 to 1000 ms (iCNV) and from −500 ms to probe onset (tCNV) were examined. Clustering in time of the preliminary significant effects was performed using an alpha level of *p* ≤ 0.05 and a channel distance of 4 cm (6.7 neighbors/channel). Second, permutation testing was performed on data clusters derived from the preliminary analyses. The permutation was executed 1000 times, and in this procedure, the data of participants were systematically interchanged, so that a new distribution of the cluster value was determined for each permutation. Based on this distribution, the probability of the initial cluster was directly determined. Only clusters that survived a statistical threshold of *p* ≤ 0.05 after correction for multiple comparisons were reported.

## 3. Results

Datasets from all participants were analyzed as no individual was deemed ineligible. The median split resulted in adolescents with lower (4.1 ± 1.7) and higher (9.1 ± 1.7) motor competencies (*F*(1, 78) = 174.7, *p* < 0.001, *η* ^2^ = 0.69). The percentage of participants in the low and high motor competence groups did not differ by gender (*χ* ^2^(1, *N* = 82) = 0.2, *p* = 0.654) and school (*χ* ^2^(2, *N* = 82) = 0.7, *p* = 0.717). Comparing both groups, there were no differences in age, height, SDQ score, and light and moderate physical activities (Table 1). In contrast, adolescents with lower motor competencies showed lower vigorous physical activity and a tendency towards a higher BMI than the other group.

### 3.1. Behavioral Performance

The uncorrected MANOVA revealed a main effect for the group on Sternberg task performance (Wilks' *λ* = 0.884, *F*(2, 79) = 5.2, *p* = 0.008, *η* ^2^ = 0.116). Including BMI and vigorous physical activity as covariates, there was also a multivariate main effect for the group (Wilks' *λ* = 0.887, *F*(2, 77) = 4.9, *p* = 0.010, *η* ^2^ = 0.113). Based on further examination, a significant univariate main effect for the group was obtained for reaction time (*F*(1, 78) = 4.7, *p* = 0.050, *η* ^2^ = 0.05), indicating shorter reaction in adolescents with higher motor competencies (816.2 ± 149.4 ms) compared to those with lower motor competencies (899.3 ± 218.8 ms). For accuracy, the univariate main effect for the group only approached significance (*F*(1, 78) = 2.7, *p* = 0.104, *η* ^2^ = 0.03). Accuracy rates were 83.6 ± 11.0% and 87.7 ± 10.9% in adolescents with low and high motor competencies, respectively.

### 3.2. Contingent Negative Variation

**Figure 1:**
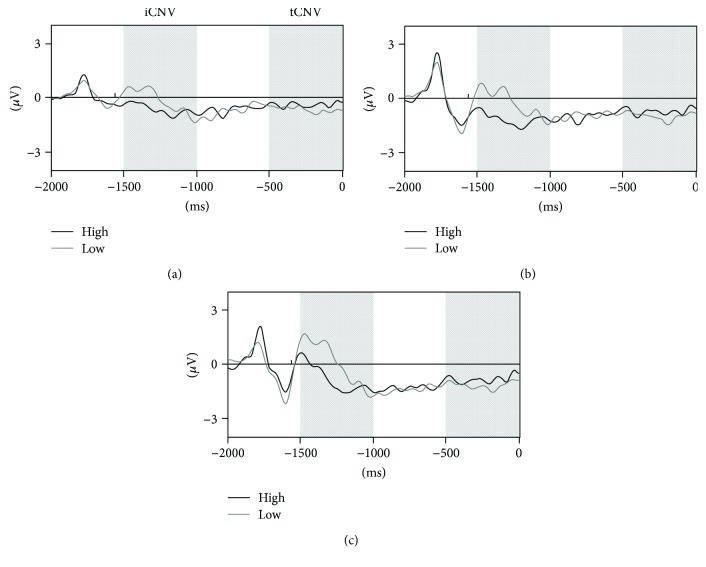
Grand averaged event-related potential waveforms at Fz (a), FCz (b), and Cz (c) within the latency range from S1 offset (−2000 ms) to S2 onset (0 ms) displayed for adolescents with low and high motor competencies. Note: the vertical bars indicate the iCNV and the tCNV.

**Figure 2:**
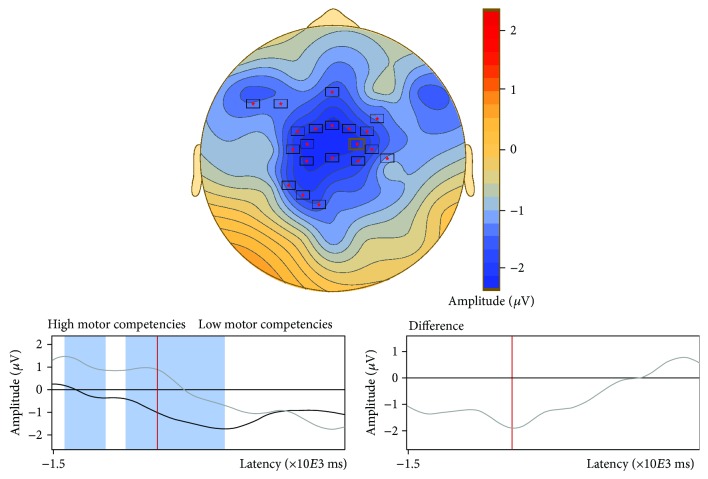
Comparison of the iCNV component (−1500 to −1000 ms) of event-related potentials between adolescents with low and high motor competencies using cluster-based permutation testing. Note: stars indicate a cluster showing significant differences between groups in a given latency range; negative values denote greater negative amplitudes in adolescent with low motor competencies compared to those with high motor competencies.

**Figure 3:**
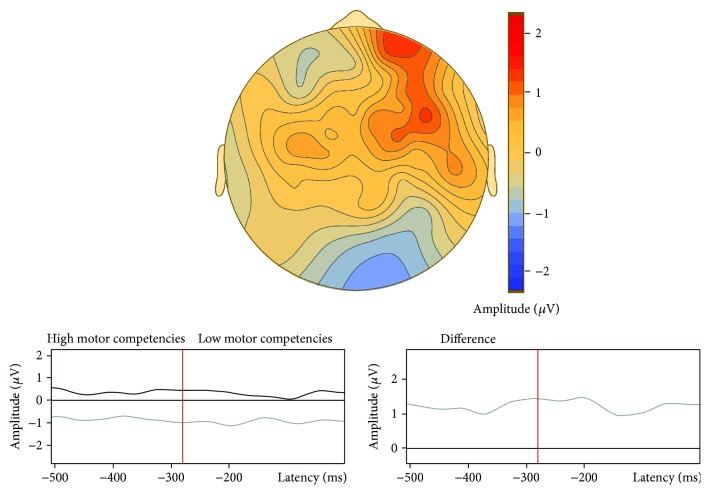
Comparison of the tCNV component (−500 ms to S2 onset) of event-related potentials between adolescents with low and high motor competencies using cluster-based permutation testing. Note: there was no significant difference between groups in the specified latency range; negative values denote greater negative amplitudes in adolescents with low motor competencies compared to those with high motor competencies.

There was no difference in the number of segments included for analysis between adolescents with high (41 ± 10; 47.8 ± 11.4% of all trials) and low motor competencies (43 ± 9; 51.4 ± 10.8% of all trials). With regard to CNV, stimulus-locked ERP waveforms for each group are displayed in Figure 1. Preliminary analyses using cluster-based permutation testing in the latency ranges from −1500 to −1000 ms and from −500 ms to S2 onset revealed no significant effects of gender, BMI, vigorous physical activity, and SDQ total score on iCNV and tCNV (see Supplemental Figure (available here)). Consequently, these variables were not entered as covariates in subsequent analyses. In comparing ERP waveforms within the iCNV time window (−1500 to −1000 ms), a significant group difference was found for a latency range from −1480 to −1148 ms (cluster value = −2114.98, *p* = 0.022), most pronounced for the frontocentral region (Figure 2). This indicated higher amplitudes in adolescents with high motor competencies compared to those with low motor competencies. For the tCNV time window (−500 ms to S2), cluster-based permutation testing revealed no differences between those groups (Figure 3).

## 4. Discussion

Based on the present results, adolescents with high motor competencies showed a greater behavioral performance on the Sternberg task compared to those with low motor competencies. This difference in working memory maintenance was indexed by a lower reaction time in high performers on MOBAK-5 tests. With regard to ERPs, a novel finding was that the groups also differed by task preparation processes. Greater iCNV amplitudes were found in adolescents with higher motor competencies compared to peers with lower motor competencies. In contrast, no differences between groups were found for tCNV.

### 4.1. Behavioral Performance

The observation that working memory maintenance was higher in adolescents with high motor competencies supports the view that there is an association between qualitative aspects of motor skills and higher-order cognition [15]. Based on a review of cross-sectional evidence, van der Fels et al. [16] suggested that this link is more pronounced in children compared to adolescents. However, this might be due to the fact that the majority of studies have examined the relationship between motor skills and higher-order cognition in children [18, 19]. The present findings expand the previous research by showing that adolescents with high performance on tasks capturing motor competencies also have high working memory maintenance. This provides some indication that qualitative aspects of motor skills are related to working memory even if the executive function system is close to maturation [14], although it has been suggested that motor and cognitive skills develop in different rates during adolescence [16].

Given that the groups differed by reaction time only, this cannot be explained by a speed-accuracy trade-off. Consequently, the observed effects were not due to group-specific strategic inclinations towards either prevention or promotion focus. It is more likely that adolescents with high motor competencies showed higher performance on the Sternberg task than those with low motor competencies, because the MOBAK-5 test battery placed similar demands on working memory maintenance. Both tasks required monitoring, planning, and sequencing of actions. These cognitive processes depend on working memory capacity and contribute to goal-directed behavior and movement [1, 11]. Thus, it appears that such higher-order cognitive processes and motor skills share common neural substrates. In this respect, an increased activation of overlapping structures has been reported with higher task complexity [15]. The MOBAK-5 test battery includes difficult locomotor and object control tasks, which place high demands on motor planning and sequencing in particular. As participants had more than one attempt, there was a chance to update motor control for subsequent movements based on the processing of motor error information [43]. Working memory resources are relied upon both planning and replanning of complex movements [44]. The observation that high motor competencies were linked with high working memory might therefore be due to the engagement of this higher-order cognitive function in the preparation and execution of movements as well as in the refinement of movement patterns for the second attempt.

### 4.2. Contingent Negative Variation

On a neurocognitive level, differences in task preparation between groups were examined via CNV. Based on the investigation of its early and late components, greater amplitude of the iCNV was found in adolescents with high motor competencies compared to those with low motor competencies. Previous experimental studies also found a decreased reaction time and/or increased accuracy on the Sternberg task along with increased iCNV, but not tCNV [22]. This provides some indication that increased behavioral performance is linked with better response orientation rather than with improved stimulus anticipation. According to Segalowitz et al. [24], the orienting response is sensitive to developmental processes and small iCNV amplitudes are suggested to reflect an immaturity of the prefrontal cortex system. From a developmental perspective, increased iCNV in adolescents with high motor competencies may indicate a greater progress in the maturation of the prefrontal cortex and other structures involved in the generation of this component [29]. This may allow more effective cognitive control, so that high performers compared to low performers on MOBAK-5 may prepare for a motor response in a qualitatively different way.

In this respect, the dual mechanism of control theory differentiates between proactive and reactive control strategies [45]. The latter refers to attention being recruited as a late correction mechanism, which is mobilized in a just-in-time manner. In contrast, proactive control is understood as a form of future-oriented early selection that allows the maintenance of goal-relevant information in a sustained manner. An increase in the iCNV elicited from a Sternberg task reflects more effective task preparation [24] and has therefore been interpreted as a change towards proactive control [22]. Since relying on proactive control is considered to benefit performance in working memory tasks [46], a higher iCNV in adolescents with high motor competencies indicates the selection of a more appropriate cognitive control strategy. However, proactive control is resource consuming due to the sustained and/or anticipatory activation of the (lateral) prefrontal cortex that goes along with continuous goal maintenance [45]. Despite this disadvantage, individuals with high working memory have been found to rely more on proactive than reactive control [47], probably due to the capacity to sustain this future-oriented early selection over an extended period of time. Consequently, high working memory maintenance in adolescents with high motor competencies seems to enable the optimization of preparation processes while minimizing interference from distractions by taking up and relying on a proactive control strategy. Further, efficient task preparation and high motor competencies seem to be linked since anticipatory action regulation and motor planning required in complex movement tasks, such as MOBAK-5 tests, are related to proactive control [48].

### 4.3. Limitations

Due to the cross-sectional design of the present study, causal relations between motor competencies, iCNV, and working memory maintenance cannot be inferred. However, a recent meta-analysis has shown that exercise programs that demand motor skills and/or cognitive engagement enhance working memory performance in children and adolescents [10]. Some first indications on a causal relationship are also provided by an experimental study showing improved task preparation processes following 8 weeks of combined aerobic and coordinative exercise, which included complex movement tasks [23]. Regarding the direction of the effects, it remains unclear whether or not training of complex motor skills benefits working memory or if improvements in this higher-order cognitive function result in increased motor competencies. Given the lack of MOBAK-5 norm values, the findings have to be interpreted with caution. However, the median split resulted in a group below and in a group above the average total score reported by a previous study with a large sample [37]. Another limitation of the present study is the lack of assessments of aerobic fitness, which has been shown to contribute to working memory [9]. It should be noted that motor competencies are fundamental skills that allow for engagement in physical activity programs [11], one of the aims of which is improvements in aerobic fitness. Such qualitative aspects of motor skills may be stable predictors of the developmental stage, so that they are more closely related to working memory maintenance than aerobic fitness. As vigorous physical activity is a predictor of aerobic fitness [49] and controlling the main effects for this variable did not change the results, there is some indirect support for a unique contribution of motor competencies to working memory. Moreover, the study results have to be interpreted with caution as other possible confounders, such as intelligence quotient and socioeconomic status, have not been assessed. However, it should be noted that all participants were attending private schools. Given that none of them received a scholarship, it is reasonable to suggest that the participants' guardians had a high socioeconomic status. Further support for this assumption is provided by a lack of differences in SDQ total score between groups, because psychopathology is closely related to socioeconomic status [50]. Additionally, all participants were taught on the same educational level, so that pronounced differences in the intelligence quotient between groups seem unlikely.

## 5. Conclusions

Adolescents with high motor competencies compared to peers with low motor competencies show greater performance on a cognitive task demanding working memory maintenance. This provides some indication that this higher-order cognitive function and qualitative aspects of motor skills are intertwined due to sharing common neural substrates. Furthermore, high motor competencies appear to be related to more effective preparation processes, which allow adolescents to take up and rely on a cognitive control strategy that is optimal in working memory tasks. Although longitudinal and experimental evidence is necessary to support this assumption, the promotion of high motor competencies might be an important target for exercise interventions that seek to improve working memory operations.

## Figures and Tables

**Table 1:** Comparison of anthropometric data, psychopathology, and physical activity between groups.

	Low (*N* = 18 females/23 males)		High (*N* = 16 females/25 males)		Total (*N* = 34 females/48 males)		ANOVA		
	*M*	SD	*M*	SD	*M*	SD	*F*	*p*	*η* ^2^
Age in years	11.0	1.1	11.3	1.3	11.2	1.2	1.4	0.238	0.017
Height in cm	151.5	8.2	152.6	7.7	152.1	7.9	0.4	0.532	0.005
BMI in kg/m^2^	19.0	3.9	17.7	2.4	18.3	3.3	3.2	0.078	0.038
SDQ total score	13.9	3.7	13.0	3.6	13.4	3.7	1.3	0.253	0.016
LPA in min/day	516.7	69.8	495.8	88.3	506.2	79.8	1.4	0.239	0.017
MPA in min/day	87.1	22.4	87.5	27.3	87.3	24.8	<0.1	0.956	0.000
VPA in min/day	35.2	16.2	47.9	24.9	41.5	21.8	7.4	0.008	0.085
Number of EEG segments	41.0	10.2	43.3	9.3	42.2	9.8	1.3	0.244	0.015

LPA = low physical activity; MPA = moderate physical activity; VPA = vigorous physical activity.

## Data Availability

Requests for data will be considered by the corresponding author.

## Supplementary Materials

Supplementary Materials Supplemental Figure 1: effects of gender, body mass index (BMI), age, psychopathology (SDQ total score), and vigorous physical activity on the iCNV (−1500 to −1000 ms) and tCNV (−500 ms to S2 onset) components of event-related potentials assessed with cluster-based permutation testing. Note: maps present *t*-values; no significant clusters were obtained within the specified latencies.
