# Supplementary material for: Contingent Negative Variation and Working Memory Maintenance in Adolescents with Low and High Motor Competencies
Source: Neural Plast. 2018 Apr 18;2018:9628787. doi: 10.1155/2018/9628787 (PMC5932462; doi:10.1155/2018/9628787)
Supplement: Supplementary Materials — Supplemental Figure 1: effects of gender, body mass index (BMI), age, psychopathology (SDQ total score), and vigorous physical activity on the iCNV (−1500 to −1000 ms) and tCNV (−500 ms to S2 onset) components of event-related potentials assessed with cluster-based permutation testing. Note: maps present t-values; no significant clusters were obtained within the specified latencies. [file 9628787.f1.docx]

**Online supplement**


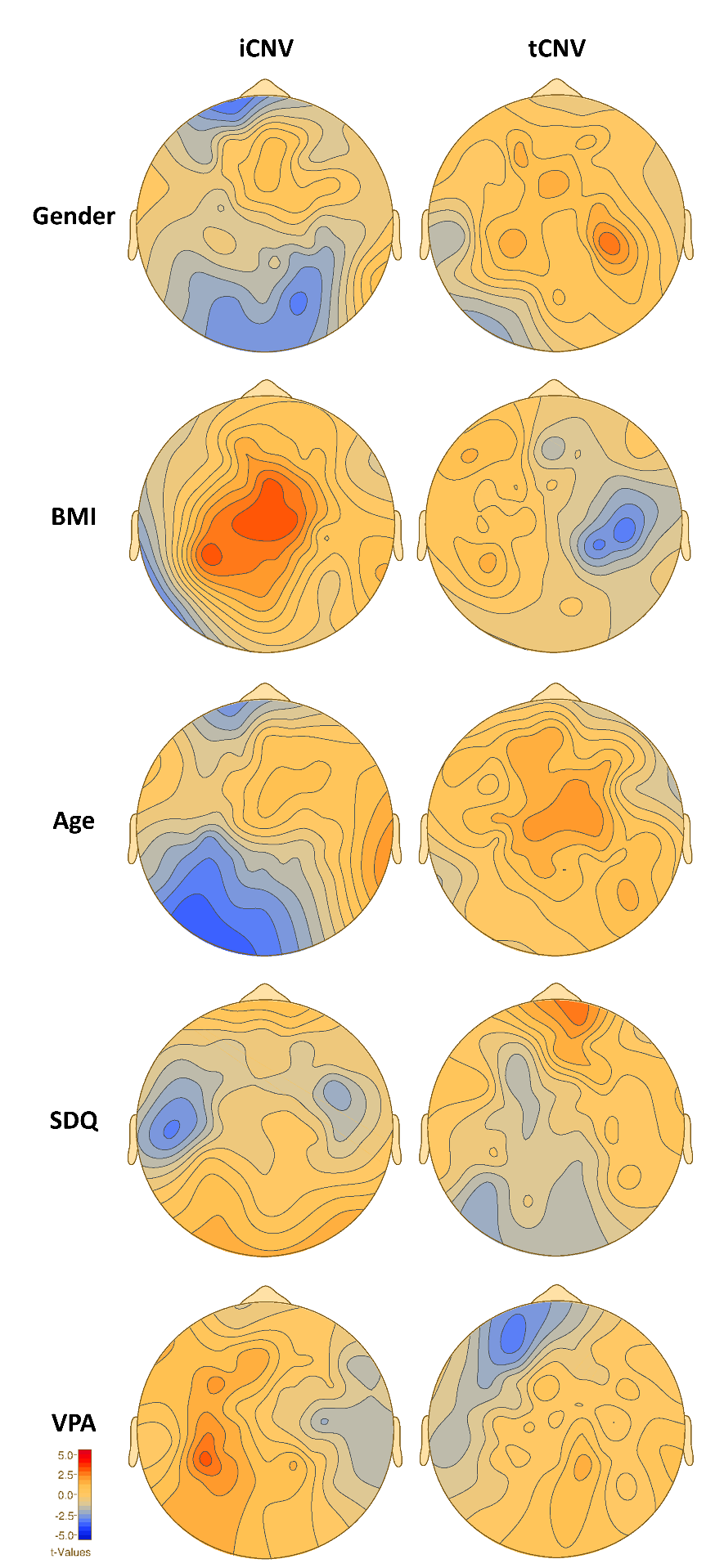


**Supplemental Figure 1.** Effects of gender, body mass index (BMI), age, psychopathology (SDQ total score) and vigorous physical activity on the iCNV (-1500 to -1000 ms) and tCNV components (-500 ms to S2 onset) of event-related potentials assessed with cluster-based permutation testing. Note: maps present t-values; no significant clusters were obtained within the specified latencies

**Additional analyses**

In the present article, motor competencies (a continuous variable) were reduced to a categorical variable using median split. Dividing particiapnts into groups with low and high motor competencies was necessary, because cluster-based permutation testing applied to the event-related potential waveforms requires the presence of at least two groups. For a high comparability with the results of the statistical analysis of event-related potential data, behavioral data was also analyzed by using group comparisons. Consequently, accuracy and reaction time were included in a single analysis as it is recommended when a positive or negative effect can appear on either reaction time, accuracy or both. However, a regression was performed to check if the reduction of the continuous variable to a categorical variable had an influence on the results. Regression-based analysis showed that the prediction of reaction time on the Sternberg task by motor competencies is significant (F=4.3; p=0.04; r=0.23), whereas it is not significant when accuracy is entered as dependent variable (F=2.9; p=0.09; r=0.19). Consequently, the outcomes of regression-based analyses corresponded to the findings obtained from MANCOVA.
